# Parotid gland pleomorphic adenoma re-operations with regard to patient and surgeon satisfaction: what can be improved?

**DOI:** 10.1080/07853890.2023.2171106

**Published:** 2023-03-07

**Authors:** Ewelina Bartkowiak, Krzysztof Piwowarczyk, Jadzia Tin-Tsen Chou, Hanna Klimza, Małgorzata Wierzbicka

**Affiliations:** 1 Department of Otolaryngology and Laryngological Oncology, Poznan University of Medical Sciences, Poznan, Poland; 2 Department of Phoniatry and Audiology, Poznan University of Medical Sciences, Poznan, Poland

**Keywords:** Oral oncology, pleomorphic adenoma, quality of life, operative report, facial nerve, contrast-enhanced MR images

## Abstract

**Background:**

Surgery, the treatment of choice for parotid pleomorphic adenoma (PA), is associated with facial nerve palsy and decreased quality of life. Re-operation for PA recurrence (rPA) significantly increases these risks and constitutes a dilemma for both patient and surgeon. Factors influencing the success of re-operation, as well as the self-reported satisfaction of both sides, have yet to be addressed in the literature. This study aims to improve upon the decision-making schedule in PA re-operations, based on patient expectations, imaging, and concordance with the first operative report (FOpR).

**Methods:**

Seventy-two rPAs treated in a single tertiary center were collected and analyzed. The FOpRs and pre-operative imaging were divided according to defined criteria into accurate and non-accurate categories. The re-operative field and course were categorized as anticipated or unanticipated. The re-operation was categorized as satisfactory or unsatisfactory for both the patient and the surgeon.

**Results:**

The accuracy of FOpRs and pre-operative imaging was 36.1% and 69.4%, respectively. Re-operative courses were: 36.1% anticipated and 63.9% unanticipated. The most frequently omitted data were: presence of satellite tumors (9.7%), and amount of removed parenchyma (9.7%). Variables that most commonly affected FOpR non-accuracy were: tumor size (Chi2(1)=59.92; *p* < 0.001) and capsule condition (Chi2(1)=29.11; *p* < 0.001). There was no significant relationship between FOpR accuracy and re-operative course (Chi2(1)=1.14; *p* = 0.286), patient satisfaction (Chi2(1)=1.94; *p* = 0.164) or surgeon satisfaction (Chi2(1)=0.04; *p* = 0.837). Pre-operative imaging (Chi2(1)=36.73; *p* < 0.001) had the greatest impact on surgeon satisfaction.

**Conclusion:**

Accurate pre-operative imaging impacted surgeon satisfaction. The impact of the FOpR on re-operation technicalities and patient satisfaction was minor. Imaging precision should be improved to streamline the decision-making process of PA re-operation. This article proposes suggestions for a future decision-making algorithm as a starting point for a prospective study.

**Key messages**
- Accurate pre-operative imaging impacts both surgeon and patient satisfaction.
- There is no significant relationship between the accuracy of the first operative report and surgeon and patient satisfaction.
- There is a statistically significant relationship between patient and surgeon satisfaction.

## Introduction

The treatment of choice for parotid gland pleomorphic adenoma (PA) is removal with intact margins and facial nerve (FN) preservation [1]. Its recurrence rate ranges from 0 to 15% [2] and depends on tumor size, presence of satellite foci, incomplete resection or capsular rupture and the patients’ age [2–4]. Parotid PAs tend to recur after long intervals, with a propensity towards multifocal disease [5]. Recurrences (rPA) are often extensive, thus burdening the second surgery with a high risk of FN damage and re-recurrence rate. There are few if any randomized studies concerning the handling of rPA, so most research conclusions appear to be based on coherent arguments. Management recommendations include observation, local or extensive surgery, radiation therapy, or a combination of the above [6–8].

While the re-operative course appears to be independent of previous actions in the operated area, subsequent surgeries nevertheless benefit from accurate imaging of the operated area and the estimated deviation from the typical treatment course [9]. The following pre-operative data can be useful: patient history including the first operative course, concordance with the first operative report (FOpR), and magnetic resonance imaging (MRI), which is the study of choice for rPA [10–12]. Accurate MRI protocols quantify: tumor spread characteristics, the number of nodules, their precise location and size, and the amount of remaining parenchyma [9]. For re-operation, the crucial datum is the contact or distance from the FN trunk and branches [13]. If the nerve was exposed and dissected, it may be very difficult to locate and preserve amongst fibrous tissue [2]. The extent of the procedure (part/whole superficial lobe/deep lobe) is less important because in the event of relapse, a radical resection is attempted with removal of residual parotid parenchyma and structures demonstrating nodular involvement. Nevertheless, this detail can guide the approach to the deep layers as well as the FN main trunk location and depth.

We wanted to maintain the hierarchy of goals: 1) to remove the tumor in its entirety; 2) to maintain the patency of the FN; 3) and, only if possible, to remove residual parenchyma with potential micro-foci. Discrepancies have been found in the pre-operative imaging, such as: size of the tumor, deep lobe extension, satellite tumors, and facial nerve retraction into the tumor; as a result, we decided to adjust our intraoperative design.

There are many discrepancies in the criteria that define different parotidectomy techniques. That is, until the European Salivary Gland Society (ESGS) introduced a new classification of salivary gland surgeries [13], specifying the level of glandular parenchyma (I–V) removed, and the non-parenchymal structures removed. A modification of parotid level I and II was proposed, marked by divisions of the FN [14]. This description is intended to optimize the management of complications. The planning of post-treatment monitoring, however, requires a good classification, a comprehensive protocol, and a clear FOpR in case of re-operation. Our work is retrospective and includes cases operated for rPA, as well as from the period before the implementation of the new parotidectomy classification in 2016. For this reason, the new classification is not included in this work.

There are no final measures of PA re-operation effectiveness except FN function and tumor recurrence. Furthermore, second surgeries are generally avoided by both patient and surgeon. Thus, we propose an assessment of both patient and surgeon satisfaction following re-operations with regard to the most optimal decision-making process, with reassurance from pre-operative data that the decision to re-operate was the right one. Precise imaging and a reliable FOpR are apparently necessary in rPA where surgical decisions are contingent upon the preceding procedure, but how do they affect the anticipated operation course and possible complications? This study intends to define inaccuracies in previous operating reports and pre-operative imaging, and to improve re-operation planning and execution. Surgeon and patient satisfaction serve as the outcome measures.

## Materials and methods

### Materials

In 2008–2017, 1154 benign parotid tumor surgeries were performed at the Department of Otolaryngology and Laryngological Surgery, a tertiary referral center. Of these, 636 (55.1%) were PAs. The recurrence rate at this institute is 6% (38/636). All primary surgeries and re-operations were performed by two experienced surgeons (MW, TK). The analysis also included patients who were initially operated for PA at a different center and were referred to our clinic for the first time for re-operation due to rPA. Therefore, the total number of re-operations due to rPA in our clinic (72) is greater than the percentage of recurrences in our department (38).

### Methods

FOpR data, histological reports and imaging examinations were reviewed before each re-operation. The following variables: sex, age, dates of first surgery and re-operation, primary operative department, amount/quality of missing data, pre-operative imaging, procedure type and histology were considered. The problem-solving analysis was based on the following criteria: FOpR accuracy, accuracy of pre-operative imaging, the anticipated operation field, and the anticipated re-operative course.

An accurate FOpR was defined as including mandatory data: patient data, surgical team, date and duration of the surgery, and procedure type. Additionally, the following seven key prognostic and anatomic elements were taken into account: tumor size, location, contact with/distance from the FN trunk or branches, presence or absence of satellite tumors, complete removal or ruptured capsule, removed parenchyma (part/whole superficial lobe/deep lobe), and post-operative FN function. A non-accurate FOpR was defined as omitting any of these seven elements.

Accurate pre-operative imaging (yes/no) was defined as including a precise description of the recurrence localization, size, character (single, multiple or miliary spread), the amount of parenchyma remaining, and concordance with operating field findings. Imaging studies had been performed in different institutions on different equipment over the last decade, and the majority of MRIs were performed according to parotid tumor protocols (multiplanar high field strength or 1.5–3 T contrast-enhanced MR images with T1 and T2-weighted sequence analyses).

An anticipated operation field was defined as a faithful reflection in the FOpR of the preserved structures and the proper amount of salivary gland parenchyma. Differences between the description and intraoperative reality classified the operation field as unanticipated.

The anticipated re-operative course is any procedure that does not meet any of the following three conditions:

1. difficulties related to unanticipated findings: scars and adhesions in the operating field, excessive bleeding, not-preserved structures, inconsistent amount of extant parenchyma;
2. discrepancies from pre-operative imaging: size, deep lobe/parapharyngeal space infiltration, satellite tumors previously not described, suspected malignancy, nerve VII retraction into the tumor and other deviations;
3. FN infiltration despite lack of clinical or radiological signs.

The occurrence of any of the above during surgery classified the operation as unanticipated.

Patient satisfaction (yes/no). After surgery, each patient was followed up in 1 month at our outpatient department. Here, the clinical examination was performed and the patient’s satisfaction was assessed. Patients were asked whether the procedure met his/her expectations concerning: aesthetics, facial muscle function, skin dysfunction, radicality of the procedure, and the need for further treatment. Patient satisfaction was categorized yes/no.

Surgeon satisfaction (yes/no). This consists of the surgeon’s honest answer *ex post* whether he/she would undertake this re-operation, or whether he/she would direct the patient for further observation or directly to radiation therapy. The surgeon selected one of two answers: ‘It is good that I undertook this surgery’ versus ‘Given better knowledge of the operational field, I would never have undertaken this surgery’.

The final measure of re-operation effectiveness was an assessment of patient and surgeon satisfaction. The primary outcome measure is the correlation between patient and surgeon satisfaction with the re-operation and examined variables, calculated as a significant relationship between satisfaction of patient and surgeon (both were dichotomous variables) versus re-operation and examined nominal variables using chi-square test. The continuous variable (age) was compared with satisfied/non-satisfied groups using t-test. The final result was the attempt to improve PA re-operation by answering the questions: which pre-operative data affected the predictability of the surgical field, which are not indispensable, and which particular parameter affected patient and surgeon satisfaction. This assessment was based on confirmed significant correlations with the primary outcome measure.

This study was approved by the Bioethics Committee of our institute (Resolution No. 781/16), and written informed consent was obtained from each patient.

### Statistical Analysis

All variables were statistically analyzed using R software, version 3.5.1. The significance level was α = 0.05. Statistical analysis was performed using the chi-squared and chi-squared test with the Yates correction for nominal variables and independent samples t-test for continuous variables (age).

## Results

### FOpR accuracy

All available FOpRs were described narratively and contained exact patient data and the operating staff roster. 36.1% (26/72) of FOpRs were classified as accurate and 52.8% (38/72) as non-accurate. Exact primary tumor data was missing in 11.1% (8/72) therefore it was not possible to classify FOpR accuracy. Data missing from the original protocols were: tumor size 4/72 (5.6%), FN contact/proximity 6/72 (8.3%), presence of satellite tumors 7/72 (9.7%), capsule condition 6/72 (8.3%), extent and amount of removed parenchyma 7/72 (9.7%), and post-operative FN function in 4/72 (5.6%) (Table 1).

**Table 1.** Distribution of the variables and data in the analyzed group.

Total patients (*N*)	72		
	***n* (%)**		
Sex:			
Male	33 (45.8)		
Female	39 (54.2)		
Primary surgical department:			
UMP	38 (52.8)		
Other	34 (47.2)		
Primary procedure:			
Extracapsular dissection	24 (33.0)		
Partial superficial parotidectomy	30 (42.0)		
Superficial parotidectomy	8 (11.0)		
Deep lobe parotidectomy	2 (3.00)		
No data available	8 (11.0)		
Re-operative procedure:			
Extracapsular dissection	23 (31.9)		
Partial superficial parotidectomy	30 (41.7)		
Superficial parotidectomy	6 (8.3)		
Total parotidectomy	1 (1.4)		
Deep lobe parotidectomy (no deep lobe parenchyma remaining)	2 (2.8)		
Selective deep lobe parotidectomy partially deep lobe parenchyma remaining)	4 (5.6)		
No data available	6 (8.3)		
	Mean ± SD (range)		
Age at first surgery	45.82 ± 15.93 (16 - 80)		
Age at re-operation	51.26 ± 15.37 (18 - 85)		
Tumor recurrence time	5.44 ± 4.21 (1.3 − 17.1)		
	Yes	No	No data available
	*n* (%)	*n* (%)	*n* (%)
Accurate FOpR	26 (36.1)	38 (52.8)	8 (11.1)
Accurate pre-operative imaging	50 (69.4)	22 (30.6)	0
Anticipated re-operative field	23 (31.9)	41 (56.9)	8 (11.1)
Anticipated re-operative course	26 (36.11)	19 (26.4)	27 (37.5)
Patient satisfaction	66 (91.7)	6 (8.3)	0
Surgeon satisfaction	48 (66.7)	24 (33.3)	0
Variables available from FOpR:			
Tumor location	68 (94.4)	4 (5.6)	0
Tumor size	68 (94.4)	4 (5.6)	0
Amount of removed parenchyma	65 (90.3)	7 (9.7)	0
Presence of satellite tumors	65 (90.3)	7 (9.7)	0
Distance from the FN trunk	66 (91.7)	6 (8.3)	0
Capsule condition	66 (91.7)	6 (8.3)	0
Postoperative status of the FN	68 (94.4)	4 (5.6)	0

The most common primary surgery was partial superficial parotidectomy and these protocols lacked: tumor size (3/30), FN contact (4/30), presence of satellite tumors (4/30), capsule condition (3/30), extent and amount of removed parenchyma (3/30), and post-operative FN function (3/30) (Table 1).

### Accurate pre-operative imaging

About 69.4% (50/72) of pre-operative imaging were classified as accurate and 30.6% (22/72) as non-accurate (Table 1). Non-accurate imaging protocols lacked: location (14/22), tumor spread characteristics (16/22), size (12/22) and amount of remaining parenchyma (20/22) (Table 2). There was also a significant relationship between the accuracy of pre-operative imaging and patient satisfaction (χ²(1)=11.52; *p* < 0.001) as well as surgeon satisfaction (χ²(1)=36.73; *p* < 0.001) (Table 2). Both patient and surgeon satisfaction were more frequent in groups with accurate pre-operative imaging versus non-accurate imaging (100.0% vs. 72.7% for patient satisfaction and 90.0% vs. 13.6% for surgeon satisfaction, respectively) (Table 2).

**Table 2.** The interdependence between the pre-operative work-up and operating field concordance, patient and surgeon satisfaction.

Variable	Accurate			Accurate			Anticipated			Anticipated			Patient satisfaction			Surgeon satisfaction		
	first operative report			pre-operative imaging			operative field			re-operative course								
	Yes (*n* = 26)	No (*n* = 38)	*p* Value	Yes (*n* = 50)	No (*n* = 22)	*p* Value	Yes (*n* = 23)	No (*n* = 41)	*p* Value	Yes (*n* = 26)	No (*n* = 46)	*p* Value	Yes (*n* = 66)	No (*n* = 6)	*p* Value	Yes (*n* = 48)	No (*n* = 24)	*p* Value
Age (mean ± SD)	48.69 ± 9.62	47.68 ± 17.83	0.771	47.54 ± 17.80	41.91 ± 9.79	0.090	48.83 ± 9.94	47.68 ± 17.25	0.738	47.96 ± 15.84	44.61 ± 16.03	0.395	46.24 ± 16.24	41.17 ± 12.12	0.459	48.35 ± 17.82	40.75 ± 9.69	0.022
Sex, Female (%)	57.7	47.4	0.578	50.0	63.6	0.416	65.2	43.9	0.169	65.4	47.8	0.234	57.6	16.7	0.134	52.1	58.3	0.802
Tumor size^a^ (%)	100.0	0.0	<0.001	40.4	52.4	0.514	100.0	7.3	<0.001	58.3	36.4	0.137	41.3	80.0	0.226	41.3	50.0	0.679
Tumor location^a^ (%)	100.0	57.9	<0.001	78.0	77.3	>0.999	100.0	61.0	<0.001	80.8	76.1	0.870	77.3	83.3	>0.999	79.2	75.0	0.920
Amount of removed parenchyma^a^ available (%)	100.0	28.9	<0.001	55.3	66.7	0.583	100.0	34.1	<0.001	72.7	51.2	0.161	56.7	80.0	0.586	54.5	66.7	0.510
Presence of satellite tumors^a^ (%)	100.0	47.4	<0.001	67.4	73.7	0.838	100.0	51.2	<0.001	76.2	65.9	0.581	67.8	83.3	0.748	68.2	71.4	>0.999
Distance from the FN trunk^a^ (%)	100.0	44.7	<0.001	69.6	65.0	0.938	100.0	48.8	<0.001	72.7	65.9	0.779	66.7	83.3	0.707	68.2	68.2	>0.999
Absence/rupture of the capsule^a^ (%)	100.0	28.9	<0.001	57.4	63.2	0.880	100.0	34.1	<0.001	68.2	54.5	0.426	57.4	80.0	0.606	58.7	60.0	>0.999
Postoperative status of the FN^a^ (%)	100.0	34.2	<0.001	63.8	61.9	>0.999	100.0	39.0	<0.001	73.9	57.8	0.299	61.3	83.3	0.531	62.2	65.2	>0.999
Procedure Type																		
Extracapsular dissection (%)	16.7	0.0	0.227	0.0	16.7	0.138	22.2	0.0	0.143	16.7	0.0	0.196	8.7	0.0	0.802	0.0	15.4	0.184
Partial superficial parotidectomy (%)	83.3	90.9		100.0	75.0		77.8	92.9		83.3	92.9		87.0	100.0		100.0	76.9	
Total parotidectomy (%)	0.0	9.1		0.0	8.3		0.0	7.1		0.0	7.1		4.3	0.0		0.0	7.7	
Accurate operative report, yes (%)	–	–	–	39.1	44.4	0.916	100.0	7.3	<0.001	52.4	34.9	0.286	37.3	80.0	0.164	38.6	45.0	0.837
Accurate pre-operative imaging, yes (%)	69.2	73.7	0.916	–	–	–	73.9	70.7	>0.999	76.9	65.2	0.442	75.8	0.0	<0.001	93.8	20.8	<0.001
Anticipated operative field, yes (%)	88.5	0.0	<0.001	37.0	33.3	>0.999	–	–	–	52.4	27.9	0.101	35.6	40.0	>0.999	36.4	35.0	>0.999
Anticipated re-operative course, yes (%)	42.3	26.3	0.286	40.0	27.3	0.442	47.8	24.4	0.101	–	–	–	39.4	0.0	0.139	37.5	33.3	0.931
Patient satisfaction, yes (%)	84.6	97.4	0.164	100.0	72.7	<0.001	91.3	92.7	>0.999	100.0	87.0	0.139	–	–	–	100.0	75.0	0.002
Surgeon satisfaction, yes (%)	65.4	71.1	0.837	90.0	13.6	<0.001	69.6	68.3	>0.999	69.2	65.2	0.931	72.7	0.0	0.002	–	–	–

Dependence between variables was assessed using chi-square test for nominal variables and t-test for continuous variables (age).

^a^This variable does not concern the exact amount of removed parenchyma, but is a 0–1 variable denoting availability of information about the removed parenchyma for each patient (i.e. 1 = information on the amount of removed parenchyma was available; 0 = information on the amount of removed parenchyma was not available.

### Anticipated operative field

About 31.9% (23/72) of re-operative fields were anticipated and 56.9% (41/72) were non-anticipated. The operation field in 11.1% (8/72) cases could not be defined because of omitted information (Table 1). Re-operations performed in anticipated re-operative fields are: partial superficial parotidectomy—7/23 (30.4%), extracapsular dissection—7/23 (30.4%), superficial parotidectomy—2/23 (8.7%), deep lobe parotidectomy—2/23 (8.7%) and selective deep lobe parotidectomy—4.3% (1/23) (Table 2). An anticipated re-operative field was observed in 100% (23/23) of accurate FOpRs (χ²(1)=48.70; *p* < 0.001). The anticipated versus non-anticipated operating field showed interdependence with: tumor size (χ²(1)=48.70; *p* < 0.001), location (χ²(1)=9.98; *p* = 0.002), amount of removed parenchyma (χ²(1)=23.57; *p* < 0.001), presence of satellite tumors (χ²(1)=14.13; *p* < 0.001), distance from the FN trunk (χ²(1)=15.29; *p* < 0.001), capsule absence (χ²(1)=23.57; *p* < 0.001) and post-operative FN status (χ²(1)=20.52; *p* < 0.001) (Table 2).

### Anticipated re-operative course

About 36.11% (26/72) of re-operative courses were anticipated: 10/26 (38.5%) extracapsular dissections, 9/26 (34.6%) partial superficial parotidectomies, 4/26 (15.4%) superficial parotidectomies, and 2/26 (7.7%) selective deep lobe parotidectomies. About 63.9% (46/72) of re-operative courses in our department were unanticipated (Table 2).

The impact of FOpR accuracy on the re-operative course was analyzed. An anticipated re-operative course followed 42.3% (11/26) of accurate and 38.5% (10/26) of non-accurate FOpRs. An unanticipated re-operative course followed 32.6% (15/46) of accurate and 60.9% (28/46) of non-accurate FOpRs. There is no statistically significant relationship between the anticipated re-operative course and FOpR accuracy (χ²(1)=1.14; *p* = 0.286) (Table 3). The anticipated versus non-anticipated operative course showed no significant interdependence with other variables (Table 2).

**Table 3.** Anticipated and unanticipated re-operative course.

Re-operative course (72)			
Anticipated		Unanticipated	
(26/72) 36.1%		(46/72) 63.9%	
Accurate FOpR	Non-accurate FOpR	Accurate FOpR	Non-accurate FOpR
(11/26) 42.3%	(10/26) 38.5%	(15/46) 32.6%	(28/46) 60.9%
χ²(1)=1.14, *p* = 0.286			

### Patient satisfaction

About 91.7% (66/72) of patients were satisfied with re-operation results while 8.3% (6/72) were not (Table 1). About 75.8% (50/66) of satisfied patients had accurate pre-operative imaging while none of the non-satisfied patients did. There is a significant relationship between patient satisfaction and accurate pre-operative imaging (χ²(1)=11.52; *p* < 0.001). Patient satisfaction was significantly correlated with surgeon satisfaction (χ²(1)=10.02; *p* = 0.002). In 72.5% of satisfied patients, surgeons were satisfied too, while in the group of non-satisfied patients, there were no satisfied surgeons (Table 2).

### Surgeon satisfaction

The surgeon was satisfied with 66.7% (48/72) of procedures, and unsatisfied in 33.3% (24/72) (Table 1). About 20.8% (5/24) of unsatisfactory surgeries had inaccurate pre-operative imaging. There is a statistically significant relationship between surgeon satisfaction and accurate pre-operative imaging (χ²(1)=36.73; *p* < 0.001), with 93.8% (45/48) of satisfied surgeons operating with accurate pre-operative imaging. In 37.5% (18/48) of procedures, the surgeon was satisfied with the anticipated re-operation course and there was no statistically significant relationship (χ²(1)=0.01; *p* = 0.931). When the surgeon was satisfied, all patients (48/48) were satisfied too. About 75% (18/24) of patients were satisfied although the surgeon was not, and 25% (6/24) of patients were not satisfied when the surgeon also declared dissatisfaction. Nevertheless, there is a statistically significant relationship between patient and surgeon satisfaction (χ²(1)=10.02; *p* = 0.002). Patients with a satisfied surgeon were significantly older (48.45 ± 17.82 years) than patients with an unsatisfied surgeon (40.75 ± 9.69 years), t(69)=2,34; *p* = 0.022 (Table 2).

## Discussion

This analysis aimed to improve the parotid tumor re-operative decision-making process through a series of questions: which pre-operative data affect surgical field predictability, which data are not indispensable, and which particular parameters affect patient and surgeon satisfaction.

Parotid tumor re-operations are characterized by technical difficulty, high morbidity, and low confidence of success. When faced with a benign parotid tumor relapse, the re-operation must be based on clinical presentation (palpation, ultrasonography, CT, and/or MRI), while data from the previous operation should provide information about the probability of tumor seeding, if only to increase the frequency of follow-up [2,9,14].

Surgeon satisfaction with the surgery was based on an honest answer *ex post* about whether he/she would undertake this re-operation. We showed that surgeon satisfaction was statistically influenced by accurate pre-operative imaging. MR imaging in our patients had been performed with variable protocols over 10 years, and it is difficult to unify this heterogeneity before re-operation. Each MRI description was compared with operative field findings. Non-accurate imaging lacked the following data: location, tumor spread characteristics, size, and amount of remaining parenchyma. The significance of thorough and adequate imaging is evident [11,15–18]. The evidence-based recommendation is multiplanar high-resolution MRI. PA lesions are well-demarcated on MRI T2-weighted sequences, demonstrating a hyper-intense signal, elevated apparent diffusion coefficient and a low intensity peripheral rim [15,19]. 3 T MRI sequences are also necessary for the accurate identification of parotid ducts, FN branches and their relation to the tumor [20,21].

Patient satisfaction was based on an honest response *ex post*: whether the procedure met his/her expectations, including aesthetics, facial muscle function, skin dysfunction, radicality of the procedure, and the need for further treatment. Surprisingly, there was also a significant relationship between the accuracy of pre-operative imaging and patient satisfaction. It is difficult to explain whether this was indirectly influenced by the surgeon’s co-satisfaction, or whether the patient compared the imaging description to the procedure description, and found their compliance satisfactory.

Patients were more often (66) satisfied than surgeons with the re-operative results (48), although a positive correlation was observed and patient satisfaction was always parallel with that of the surgeon. When the surgeon was satisfied after the procedure, all patients were satisfied too.

There was no significant relationship between FOpR accuracy and surgeon satisfaction. We have focused on the validity of operating reports, assuming they would be helpful in everyday practice, especially in designated salvage institutions. There was no literature about how the application of an operative classification with a detailed FOpR impacted re-operation planning. Our analysis was carried out in 2017, so none of the FOpRs incorporated the new ESGS classification. Our single institution analyses of re-operations revealed that because of scant information in narrative FOpR, some information was not available for decision-making and guidance during re-operation. To summarize, the simple, comprehensive classification presented by ESGS is not adequate for tertiary referral departments that deal with failure cases. Furthermore, implementations such as the ESGS parotid gland surgery classification are not readily accepted by otolaryngologists, and their incorporation into practice takes time [22]. Although we did not find any correlation between the FOpR and surgeon satisfaction, the anticipated versus non-anticipated operating field showed interdependence with missing data concerning tumor size and location, amount of removed parenchyma, presence of satellite tumors, distance from the FN trunk, capsule absence and post-operative FN status. Presumably the shortcomings of the first operating protocols were compensated by exhaustive imaging descriptions.

From this, we propose that parotid gland OpR should include more mandatory data: tumor size (<2 cm/2–4 cm/>4 cm), location (according to the ESGS), FN contact (main trunk, branches), presence or absence of satellite tumors, complete removal or ruptured tumor capsule, removed parenchyma (part/whole superficial lobe/deep lobe), post-operative FN function (intact or injured), and intraoperative difficulties (scars, adhesions, and excessive bleeding).

In this cohort of re-operative PA patients, a few words should be said with regard to postoperative FN paralysis or paresis. None of the 38 patients operated in our Department developed FN palsy as a result of their primary procedure. After re-operation of rPA, 4 (11%) developed paresis of the FN marginal branch. In contrast, 14 out of 34 (41%) patients operated outside our Department had FN palsy. After re-operation of rPA, FN palsy developed *de novo* in 2 patients, and worsened in 10 of the 14.

Our analysis is unique in many aspects. In the literature, there are no clear indications, recommendations or long-term analyses of the results of PA re-operation despite experienced surgeons knowing these facts, traps and clues very well. To our knowledge, this is the first study to quantify reasoning for surgeon satisfaction in PA re-operation. The most important parameter impacting patient and surgeon satisfaction is pre-operative imaging, and we strongly recommend the best possible MRI quality, especially in the case of referred patients. In all cases, we recommend obtaining full documentation of the first operation.

Our study is limited by its retrospective nature and the inability to retrieve the data that would otherwise complete the patient satisfaction survey; thus, patient satisfaction was obtained during the first post-operative follow-up in the outpatient department. Hence, we do not know how it was shaped by the following criteria: aesthetics, facial muscle function, skin dysfunction, radicality of the procedure, and the need for further treatment. Nevertheless, the use of this annotation in the outpatient card allowed for a concise categorization of the patient’s re-operative assessment.

## Conclusions

Accurate pre-operative imaging impacted surgeon satisfaction. The impact of the FOpR on the technical aspects of the re-operation and patient satisfaction is minor. There is no final decision-making algorithm as this is a preliminary study, but because of the considerable number of patients in our cohort, we have implemented suggestions for future decision-making. Imaging precision should be improved to streamline the decision-making process of parotid tumor re-operation. This article with the aforementioned schedule is a starting point for a prospective study.

## Data Availability

The authors confirm that the data supporting the findings of this study are available within the article.
